# Effect of Psychosocial Interventions for Individuals Who Underwent Arthroscopy in Femoroacetabular Impingement: A Randomized Controlled Trial

**DOI:** 10.3390/jcm12113612

**Published:** 2023-05-23

**Authors:** Mingke You, Shuoyao Yang, Jian Li, Gang Chen

**Affiliations:** Department of Orthopedics, Orthopedic Research Institute, West China Hospital, Sichuan University, Chengdu 610041, China

**Keywords:** hip joint, arthroscopy, femoroacetabular impingement syndrome, psychosocial intervention, rehabilitation

## Abstract

Purpose: The purpose of this protocol was to discover the connection between patients with non-surgical pain or other discomfort and their psychosocial status. Cognitive behavior therapy will be used, which we verified will determine the effect and feasibility of postoperative rehabilitation processes. Materials and Methods: This study will include 200 patients ranging from 18 to 60 years old who have underwent or will undergo FAI arthroscopy in the West China Hospital Sports Medicine Center from 2023 to 2026. A standardized prospective single-center parallel-group randomized controlled trial will be used for these participants. The participants will be divided into intervention (telephone versus face-to-face versus music versus floatation) and control groups. The follow-up periods will be measured pre-operatively, as well as postoperatively at 1, 3, and 6 months. The primary outcomes will include the modified Harris Hip Score (mHHS) and the Visual Analogic Score (VAS), and the secondary outcomes will include the range of motion (ROM), the Huaxi Emotional-distress Index (HEI), and the depression, anxiety, and stress scale (DASS-21 scale). Furthermore, the Patient Health Questionnaire-9 (PHQ-9) and a Short-Form 12 (SF-12) questionnaire will also be evaluated. Discussion: This study will evaluate the clinical and cost-effectiveness of different types of psychosocial-therapy-based rehabilitation methods designed to improve the quality of life of FAI patients with persistent symptoms.

## 1. Introduction

Femoroacetabular impingement (FAI) is one of the clinical syndromes characterized by femoral head or/and acetabulum anatomic abnormalities that mostly occurs in young adults, especially young athletes, accompanied by symptoms of gait abnormality, groin pain, or mobility limitation. To treat FAI, hip arthroscopy plays an important role in modern hip-preserving surgery, with minimal invasiveness, fast recovery time, and a low rate of complications [1,2]. Furthermore, its postoperative benefits are considerable, including an increased length of stay and decreased pain. The common factors that affect its outcomes include surgical technique, rehabilitation, and the patient’s psychological state. Among these, psychological factors may influence a patient’s postoperative outcomes the most, determining whether they return to sport.

Adverse effects related to psychological factors, including anxiety, pain, and delirium management, together with narcotics-related complications and other postoperative symptoms, may result in more severe conditions, such as morbidity and mortality [3,4]. Although some researchers have verified that pain before surgery occurs more often than post-operation, studies on the postoperative pain and discomfort of angiogenic factors have also been reported, especially at 3, 6, and 12 months after surgery [5,6]. However, on the other hand, an insufficient quantity of research suggests a lack of cases, and no standardized approach to pain management has been summarized.

Improvements in psychosocial factors are strongly related to a participant’s quality of life and activity abilities. Waerden et al. conducted an exercise and a psycho-education program and collected the short- and long-term outcomes. They found that exercise combined with psycho-education may be an option for depression prevention in women with low levels of education [7]. Hu et al. conducted research on floatation therapy in the Chinese population and found that it provided a deepened state of relaxation, reducing stress levels and improving subjects’ emotional states [8].

Additionally, patients after total hip or knee arthroplasties with infection suffered from psychological burdens, implying a correlation [9]. Bay et al. performed a systematic psychological evaluation of total hip arthroplasty (THA) patients and found that psychological interventions were not routinely applied for THA; therefore, determining the applicable conditions for and optimal usage of THA is still required [10]. Additionally, there were no comparisons between different psychosocial interventions in FAI patients in the literature.

Therefore, this trial’s primary objective is to compare the effectiveness of different psychosocial therapies for improving the quality of life of FAI participants with persistent postoperative symptoms. The secondary objective is to determine the relationship between clinical effectiveness, patients’ feedback, and relative cost-effectiveness.

## 2. Materials and Methods

### 2.1. Study Design and Recruitment

**Figure 1:**
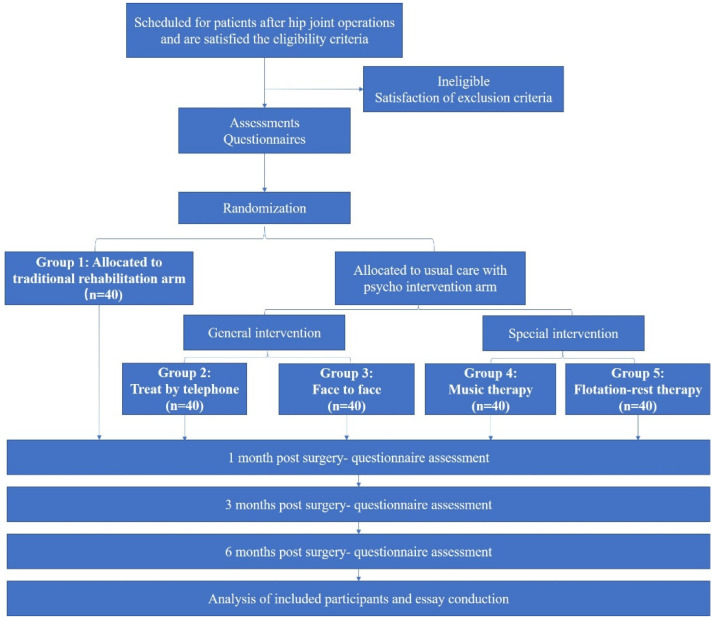
A flow diagram of the study protocol.

A parallel-group randomized controlled trial will be conducted according to the Standard Protocol Items: Recommendations for Interventional Trials (SPIRIT) checklist. Participants will be asked to sign the informed consent document before treatment. Then, we will randomize them into either the intervention or control group before treatment. The educational intervention will be the same in both groups. An examination of patients’ feedback, as well as a relative cost-analysis, will be performed at the last follow-up. Detailed information is presented in Figure 1.

### 2.2. Eligibility Criteria

The inclusion criteria include the following: (1) initially diagnosed with FAI and received treatment by arthroscopy; (2) positive postoperative radiological diagnosis of hip joint rehabilitation; and (3) anxiety and depression symptoms on the HEI scale at pre-operation was ≥8.

The exclusion criteria include the following: (1) patients with severe psychiatric disorders requiring medical treatment or other interventions; (2) revision surgery; and (3) participants who meet the contraindications according to the specific therapeutic intervention program.

Ethics approval was attained from the West China Hospital Ethics Committee; written consent will be provided by each individual.

### 2.3. Randomization and Allocation Concealment

The randomization schedule will be completed by the study biostatistician’s computer-generated random numbers table. The allocation ratio will be generally equal for patients allocated in the five groups mentioned above. Participants and physiotherapists will both receive random numbers sealed in envelopes. The matching envelopes will be allocated and stored by a researcher not participating in any recruitment, treatment, or assessment process.

### 2.4. Blinding

Patients or physiotherapists cannot be blinded during the postoperative treatment of psycho-related content in these specific educational contexts. Conversely, researchers that guide the patients to complete the data will be blinded, as will be the data statistician responsible for analyzing the statistics and data outcomes.

### 2.5. Baseline Data Collection

The genders, ages, occupations, durations of pain or other discomfort, medications, surgical and psychosocial interventions, surgical records, and scanning of images will be recorded.

### 2.6. Intervention

All patients diagnosed with FAI have already undergone hip arthroscopy with debridement or the repair of labral tears, and the postoperative rehabilitation would have been conducted at West China Hospital. Patients in the TAU or traditional rehabilitation group will be applied to a semi-structural rehabilitation program with modified exercise prescriptions according to the individual’s assessment.

Patients will be instructed to perform isometric contractions within one day before the surgery. After two weeks, partial weight bearing with assistive devices, including crutches and braces, will be recommended, together with passive range-of-motion and anterior capsule stretching exercises [7,8]. The hip joint should not be performed external rotation at leass than three weeks postoperatively [9]. Within 4–8 weeks after surgery, the extent of weight bearing will be decided by the patient’s endurance during rehabilitation, with practices, including muscle strength, balance, and cardiorespiratory exercises. In addition, posterior capsule stretches will be conducted gradually. Sports activities will be advised at 6–8 weeks after surgery, with a return to sports achieved after approximately 10 weeks of rehabilitation [9,10].

## 3. Psychological Intervention

### 3.1. Educational Intervention

The participants will receive different psychoeducation programs according to their allocation. All subgroups will receive treatment, including cognitive behavioral therapy and progressive muscle relaxation intervention, during the program [11]. Each session will last for 20–30 min, and the patients will receive a semi-structured dialogue together with brief written advice (BWA) adjusted according to their contemporary situation [12]; furthermore, positive feedback will be provided after each treatment [13]. One session will be conducted per week for six weeks, and the whole evaluation process will last for 3–6 months, with assessments covered in every follow-up. Inpatient therapy will be conducted during patients’ stay at the hospital, while for patients after discharge, therapy will be conducted through at-home training or outpatient sessions [14,15,16]. The purpose of the whole intervention is to achieve emotional relief and self-relaxation.

The educational program will be conducted face-to-face (Group 2) or by telephone (Group 3) according to the participant’s actual condition, including transportation inconveniences. Before the psychological intervention, stretching exercises will be conducted for relaxation in all subgroups [13]. The psychoeducation contents in each group will be much the same, with a semi-structured dialogue given by the nurses or physiotherapists, with necessary guidance and contents, including patient experience sharing, situation sharing that may cause depression and stress, information sharing, problem-solving, skill training, etc. This ensures dialogue completeness, with roughly the same contents and consistency for each group [12,17,18]. In particular, information about pain and sleep management skills; advice on gradual build-up activities, as well as the pace and consistency of activity; and explanations of rest and conditioning will be provided in detail [19,20].

Furthermore, both groups will be efficiently verified based on previous research [21,22], while the discrepancy between group 2 and group 3 would mainly lie in experience sharing, in which team sharing is expected to have a better result due to the potential situational entrapment while communicating and sharing. One expected difference between the reported face-to-face group and the telephone group is that the latter may have difficulties in understanding certain aspects due to the lack of facial and visual cues [17,23].

### 3.2. Therapeutic Intervention

Two therapeutic methods will be conducted separately with the same curation frequency and period. Unlike the educational intervention, the therapeutic intervention will cover music therapy and flotation-REST therapy to provide deeper relaxation by offering a more creative environment for participants to engage in symbolic therapeutic activities, such as sensory isolation, to reduce stress and other negative expressions. Although face-to-face conditions may also occur in the two therapeutic groups, we explored the therapeutic effects of music or lying on sand rather than face-to-face communication.

Music therapy (group 4): This therapy involves three parts: A Type I song, a therapeutic song, and a Type II song. Type I is also known as a welcome song, and will be mainly used to activate the patient’s cognitive areas and stimulate recent memories with a relatively slow rhythm. Classical or romantic pieces are usually selected for this period, which lasts for 2–4 min. The therapeutic song will be selected depending on the patient’s interests. For the last part, a progressively faster piece compared with a Type I song will be selected (above 80 bpm). Furthermore, this period would involve lyrics to stimulate the patient’s social and emotional behaviors, including self-esteem. The three musical parts maintain a consistent same volume of 50 decibels [24,25].

Flotation-REST therapy (group 5): This therapy will be conducted by an individual in a soundproof, lightproof, and temperature-controlled (35° Celsius) environment to minimize sensory signals. We need a flotation tank with reverse osmosis water saturated with Epsom salt (magnesium sulfate) to ensure that the whole body is soaked and floats. During the treatment, patients are instructed to lie horizontally and face-up to deepen their relaxation. Notably, a one-week adjustment would be provided considering female patients’ menstrual cycle periods [26,27].

### 3.3. Control

Patients in this group will receive a traditional rehabilitation program performed by therapists and will adhere to the therapeutic protocol as much as possible. Problems and issues will be solved simultaneously through discussion. In addition, the standardized, structured treatment will be recorded by researchers that have not participated in other parts of the research.

## 4. Measures

### 4.1. Outcome Measures

The modified Harris Hip Score (*mHHS*) contains eight items affiliated with three domains representing clinical aspects, including pain and functional grade (gait, activities, etc.). The mHHS will be scored from 0 (worst functional outcome and maximum pain) to 100 points (best functional outcome and least pain) for each item in the arbitrary weight. Patients with a score of <70 represent a poor result, while a score between 70 and 79 refers to a fair result, a score between 80 and 89 is good, and an excellent result requires a total score over 90 [28,29]. This measurement is used to assess the relationship between physical function and psychological conditions after hip arthroscopy, providing a high construct validity for evaluating bodily pain.

The depression, anxiety, and stress scale (DASS-21 scale) is a self-reported 21-item scale, with subscales, including depression, anxiety, and stress. Each subscale contains seven items, and each is scored from 0 (did not apply to me at all) to 3 (applies to me very much or most of the time). The final score is determined by the total sum of each item, which is subsequently multiplied by 2. For each subscale, scores over 9 represent an indication of depression, a score of 7 indicates anxiety and a score of 14 indicates stress symptoms. Traditionally, the higher the final scores, the more severe the patient’s symptoms will be [30,31,32]. Based on its low cost and ease of administering, as well as providing a higher precision for elderly patients together with reliability and validity, this questionnaire will be used to assess the subjective depressive, stress, and anxiety complaints of participants, as well as their frequency and severity [33].

Patient Health Questionnaire-9 (PHQ-9): The PHQ, which is usually used as a cutoff for diagnosis, will be developed to diagnose depressive disorders, as well as the severity of depression, through the filling out of a questionnaire containing nine items. Each item is rated on a 4-point scale, with ranges from 0 (not at all) to 3 (nearly every day). As the most used clinical diagnosis measurement, a total of 27 is possible, with higher scores illustrating a higher likelihood of severe depressive disorder symptoms [34,35]. The aspects in this questionnaire are mainly correlated to the patient’s psychosocial characteristics, cultural background, and contributing postsurgery symptoms, with a reported sensitivity of 83.84% and specificity of 97.01%, as well as a Cronbach’s α of 0.88 reported in a Norwegian-speaking community sample [36,37].

Huaxi Emotional-distress Index (HEI): The HEI is mainly used as an assessment tool for the rapid screening of inpatients’ psychological status. It covers 11 items for evaluations of depressive or anxious emotions. Each item corresponds to 4 points for the first 10 items, with effects from 0 (no influence) to 4 (severe influence). Scores below 8 indicate normal, scores above 8 indicate negative emotions, and specifically for patients who answered item 9 with 2 or above, reality verification or an assessment of suicidal thoughts may be required. If the total of the first 9 items is above 8, then continue covering items 10 and 11; otherwise, the evaluation is ended. The HEI scale is suitable for localized patient conditions, with a reported validity and reliability of 0.898 and 0.878, respectively [38,39,40,41].

As for each re-assessment of the rehabilitation process, the 6 min walk test will be taken to evaluate a patient’s walking speed, balance, coordination, and ability to perform daily activities. The detailed assessments are listed below in Table 1.

### 4.2. Adherence to the Traditional Rehabilitation Program Group

The number of visits will be recorded after treatment, together with frequency, intensity, time, training type and volume, and exercise progression, both at the ward and at home. Numeric rating scales (0 refers to not at all and 10 refers to completely as instructed) will be used to evaluate the overall adherence to the whole program, with separate components according to specific items.

### 4.3. Relative Hospitalization Cost Assessment

The relative hospitalization cost includes participant hospitalization, outpatient treatment during follow-ups, medication usage, and health services. The aim of this assessment is to determine which intervention is cost-effective for treatment.

### 4.4. Adverse Events, Medication Usage, and Co-Interventions

Adverse events and medication use will be recorded along with the follow-up process at pre-operation, and at 1, 3, and 6 months after surgery by stages. Physical activities in all subgroups will be recorded each week after timely treatment. In addition, any other special circumstances, including necessary medication usage and co-interventions (intensity, frequency, and type), will be collected and immediately recorded.

### 4.5. Sample Size

The primary endpoint will be altered from baseline to 6 months post-surgery in the mHHS scale. A pilot trial was conducted, and the baseline suggested the targeted patients’ population, with a mean mHHS of 85.1 and an SD of 8.7. The minimal clinically important difference (MCID) for the mHHS scale in this population is 8 points [29,42]. Therefore, the sample size of this study will be calculated based on the mHHS SD of 8.7 and the MCID of 8.

G*power software (version 3.1.9.7 for Windows XP, Vista, Germany) was used to calculate the sample size, with an effect size of 0.8 and power of 90%, and a sample size of 34 in each group is required for the minimum inclusion. Considering the allowance of 15% for a loss of follow-up for 12 months postoperatively, 40 participants will be recruited in each group.

### 4.6. Statistical Analysis

Statistical analysis will be conducted through IBM SPSS 22.0. The primary purpose of this protocol is to discover the connection between the relief of pain or discomfort post-operation and the medical staff’s psychological intervention. Primary outcomes will be estimated to determine the statistical significance (at a significance level of 0.05). Other outcomes and different endpoints aiming at illustrating the patient’s psychological conditions and the rehabilitation situations would also be analyzed. Descriptive analysis will be conducted to explore the effects of subgroups based on specific surgical techniques. Analysis of covariation (ANCOVA) will be used to assess changes in various time points, offering evidence at a 95% confidence interval to evaluate the effects on the final clinical result. SDs and outcomes in each time point at the mean change will comprise descriptive statistics and linear regression random-effects models will be used to describe the differences in mean changes between the groups.

As for the underlying missing data or incomplete adherence in this protocol, regardless of normal study attrition, steps to minimize the loss of follow-ups and maximize adherence to the rehabilitation program will be taken. A sensitivity analysis will be conducted to analyze the missing data and their potential effect on the final result, as well as an evaluation of the baseline comparability between the traditional rehabilitation group and the psychological intervention group. Any changes in protocol design will be recorded with reasonable justifications.

## 5. Discussion

This study provides a protocol for a randomized controlled trial to identify practical operability and clinical- and cost-effectiveness, as well as to determine participants’ satisfaction. This trial will assist in identifying the relationship between a patient’s subjective feelings and unwell symptoms, as well as the underlying psychosocial factors, by offline supervised training, therapy adjustment, and long-term follow-ups, enabling further improvements in treatment strategies.

Recent studies have shown that although pain is one of the major factors contributing to clinical outcomes and patients’ satisfaction, there is still a considerable need for high-quality RCT to develop optimal pain management strategies with minimal complications [4]. In addition, researchers have reported that psychological factors may increase pain severity [16,43,44,45]. Renee et al. [43] reported an amplification of somatic symptoms, including headache, abdominal pain, and impairment, due to depressive emotions. Furthermore, Maria et al. [44] and P. Cristancho et al. [45] also reported that increasing pain resulted from emotional dysfunction for elderly patients and patients with hip fractures. The research above supports the hypothesis that psycho-related problems result in increased severity of pain or discomfort; however, there is still a lack of high-quality evidence.

Other related studies have documented psycho-social factors affecting postoperative symptoms in other regions of the human body. Six studies reported an association between psychological factors and postoperative pain after knee surgeries [46,47,48,49,50,51]. Traditionally, psychological problems before surgery are more common than after surgery, and as for the latter period, post-operation follow-ups after 3, 6, and 12 months were primarily used for the identification [52,53]. Particularly, four of the six selected articles reported significant changes in psychological conditions when catastrophizing pain scores [46,47,50,51]. Furthermore, one merely explored the importance of psychosocial evaluation for interventions associated with pain and functional outcomes after surgery [49], while the others indicated no significant changes in the clinical outcomes [48].

The limitations of this study include the impossibility of participants and researchers being blinded in clinical trials; furthermore, the possibility of assessor bias cannot be discounted. Other factors, such as the participant’s practical adherence and completeness towards rehabilitation programs and psychological interventions should also be considered.

The innovations of this trial include: 1. this protocol is the first RCT study in psychosocial treatment for FAI participants with persistent postoperative symptoms; 2. the psychosocial treatment and staged assessment can be personalized, with therapy adjusted accordingly; and 3. this trial can guide the clinical decision making of targeted postoperative rehabilitation programs, developing indispensable interventions for future applications.

## Figures and Tables

**Table 1:** Summary of outcome assessments and data collection measures.

T0 assessment: baseline	mHHS, ROM, VAS, DASS-21, PHQ-9, HEI, SF-12
T1 assessment: 1 month	mHHS, ROM, VAS, DASS-21, PHQ-9, HEI, SF-12, clinical significance
	**plan adjustment**
	symptom↑ or participant’s feedback of heavy burden: adjust participant’s condition, decrease training volume or time smoothly, and drug usage if necessary
	symptom↓ or participants feedback positive: continue or increase training time smoothly
T2 assessment: 3 months	mHHS, ROM, VAS, 6-min walk test, DASS-21, PHQ-9, HEI, SF-12, clinical significance
T3 assessment: 6 months	mHHS, ROM, VAS, 6-min walk test, DASS-21, PHQ-9, HEI, SF-12, clinical significance, satisfaction, cost-effective analysis

mHHS: modified Harris Hip Score; VAS: Visual Analogic Score; ROM: range of motion; HEI: Huaxi Emotional-distress Index; DASS-21: depression, anxiety, and stress scale; PHQ-9: Patient Health Questionnaire-9; SF-12: Short-Form 12.

## Data Availability

Evaluation scales have been submitted in the Supplementary Materials.

## Supplementary Materials

The following supporting information can be downloaded at: https://www.mdpi.com/article/10.3390/jcm12113612/s1.

## Trial Registration

Trial registration: this study was registered on the Chinese Clinical Trial Registry (ChiCTR2000033646) on 7 June 2020.
