# Supplementary material for: Effect of Psychosocial Interventions for Individuals Who Underwent Arthroscopy in Femoroacetabular Impingement: A Randomized Controlled Trial
Source: J Clin Med. 2023 May 23;12(11):3612. doi: 10.3390/jcm12113612 (PMC10253459; doi:10.3390/jcm12113612)
Supplement: Supplementary file 1 [file jcm-12-03612-s001.zip › evaluation scales.pdf]

Modified Harris Hip Score

Pain

__None/ignores (44 points)
__Slight, occasional, no compromise in activity (40 points)
__Mild, no effect on ordinary activity, pain after activity, uses aspirin (30 points)
__Moderate, tolerable, makes concessions, occasional codeine (20 points)
__Marked, serious limitations (10 points)
__Totally disabled (0 points)

Function:

Gait limp

__None (11 points) __Slight (8 points) __Moderate (5 points) __Severe (0 points)

Support

__None (11 points)
__Cane, long walks (7 points)
__Cane, full time (5 points)
__Crutch (4 points)
__2 canes (2 points)
__2 crutches (1 points)
__Unable to walk (0 points)

Distance walked

__Unlimited (11points) __6 blocks (8 points) __2-3 blocks (5 points) __ Indoors only (2 points) __Bed and chair (0 points)

Functional activities: stairs

__Normally (4 points) __Normally with banister (2 points) __Any method (1 points) Not able (0 points)

Socks/Shoes

__With ease (4 points) __With difficulty (2 points) __Unable (0 points)

Sitting

__Any chair, 1 hour (5 points)
__High chair, 1/2 hour (3 points)
__Unable to sit, 1/2 hour, any chair (0 points)

Public transportation

__Able to enter public transportation (1 points) __Unable to use public transportation (0 points)

Range of motion (ROM)

Hip: flexion, extension, abduction, adduction, external rotation, internal rotation

Visual analogue scale (VAS)

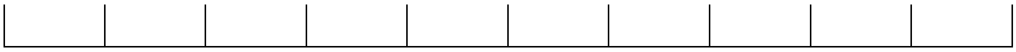

0 1 2 3 4 5 6 7 8 9 10

0 points: no pain

1~3 points: mild pain

4~6 points: moderate pain

>7 points: severe pain

Depression, Anxiety and Stress Scale (DASS-21)

Statement	Did not apply to me at all	Applied to me to some degree or some of the time	Applied to me a considerable degree or a good part of the time	Applied to me very much or most of the time
1. I found it hard to wind down	0	1	2	3
2. I was aware of dryness of my mouth	0	1	2	3
3. I couldn't seem to experience any positive feeling at all	0	1	2	3
4. I experienced breathing difficulty (eg, excessively rapid breathing, breathlessness in the absence of physical exertion)	0	1	2	3
5. I found it difficult to work up the initiative to do things	0	1	2	3
6. I tended to over-react to situations	0	1	2	3
7. I experienced trembling (eg, in the hands)	0	1	2	3
8. I felt that I was using a lot of nervous energy	0	1	2	3
9. I was worried about situations in which I might panic and make a fool of myself	0	1	2	3
10. I felt that I had nothing to look forward to	0	1	2	3
11. I found myself getting agitated	0	1	2	3
12. I found it difficult to relax	0	1	2	3
13. I felt down-hearted and blue	0	1	2	3
14. I was intolerant of anything that kept me from getting on with what I was doing	0	1	2	3
15. I felt I was close to panic	0	1	2	3
16. I was unable to become enthusiastic about anything	0	1	2	3
17. I felt I wasn't worth much as a person	0	1	2	3
18. I felt that I was rather touchy	0	1	2	3
19. I was aware of the action of my heart in the absence of physical exertion (eg, sense of heart rate increase, heart missing a beat)	0	1	2	3
20. I felt scared without any good reason	0	1	2	3
21. I felt that life was meaningless	0	1	2	3

For questions numbered 3, 5, 10, 13, 16, 17, 21 refer to depression evaluation; for questions numbered 2, 4, 7, 9, 15, 19, 20 refer to anxiety evaluation; 1, 6, 8, 11, 12, 14, 18 refer to stress evaluation.

Patients Health Questionnaire (PHQ-9)

	Not at all	Several days	More than half the days	Nearly everyday
1. little interest or pleasure in doing things	0	1	2	3
2. feeling down, depressed, or hopeless	0	1	2	3
3. trouble falling or staying asleep, or sleeping too much	0	1	2	3
4. feeling tired or having little energy	0	1	2	3
5. poor appetite or overeating	0	1	2	3
6. feeling bad about yourself—or that you are a failure or have let yourself or your family down	0	1	2	3
7. trouble concentrating on things, such as reading the newspaper or watching television	0	1	2	3
8. moving or speaking so slowly that other people could have noticed. Or the opposite—being so figety or restless that you have been moving around a lot more than usual	0	1	2	3
9. thoughts that you would be better off dead, or of hurting yourself	0	1	2	3

	Depression severity
1-4	Minimal depression
5-9	Mild depression
10-14	Moderate depression
15-19	Moderately severe depression
20-27	Severe depression

Huaxi Emotional-distress Index (HEI)

	Never	Occasionally	Some of the time	Most of the time	Nealy all the time
1. felt so depressed that nothing could cheer you up					
2. had little interest in anything					
3. felt nervous or tense					
4. worried a lot					
5. felt too uncomfortable to calm down					
6. felt suddenly scared, afraid or panicky multiple times					
7. blamed yourself frequently					
8. felt hopeless					
9. had thoughts of ending your life					

<50 points: normal

50-60 points: mild depression or anxiety

60-70 points: moderate depression or anxiety

>70 points: severe depression or anxiety

Short-Form-12 (SF-12)

1. in general, would you say your health is:
__excellent (1 points) __very good (2 points) __good (3 points) __fair (4 points) __poor (5 points)

2. does your health now limit you in moderate activities, such as moving a table, pushing a vacuum cleaner, bowling or playing golf? If so, how much?
__yes, limited a lot (1 points) __yes, limited a little (2 points) __no, not limited at all (3 points)

3. does your health now limit you in climbing several fights of stairs? If so, how much?
__yes, limited a lot (1 points) __yes, limited a little (2 points) __no, not limited at all (3 points)

4. during the PAST FOUR WEEKS, how much of the time have you accomplished less than you would like with your work or other regular daily activities as a result of your physical health
__all of the time (1 points) __most of the time (2 points) __some of the time (3 points) __a little of the time (4 points) __none of the time (5 points)

5. during the PAST FOUR WEEKS, how much of the time have you not done work or other activities as carefully as usual as a result of your physical health.
__all of the time (1 points) __most of the time (2 points) __some of the time (3 points) __a little of the time (4 points) __none of the time (5 points)

6. during the PAST FOUR WEEKS, how much of the time have you accomplished less than you would like with your work or other regular daily activities as a result of any emotional problems (such as feeling depressed or anxious)
__all of the time (1 points) __most of the time (2 points) __some of the time (3 points) __a little of the time (4 points) __none of the time (5 points)

7. during the PAST FOUR WEEKS, how much of the time have you not done work or other activities as carefully as usual as a result of any emotional problems (such as feeling depressed or anxious)
__all of the time (1 points) __most of the time (2 points) __some of the time (3 points) __a little of the time (4 points) __none of the time (5 points)

8. during the PAST FOUR WEEKS, how much did pain interfere with your normal work (including both work outside the home and housework)
__not at all (1 points) __a little bit (2 points) __moderately (3 points) __quite a bit (4 points) __extremely (5 points)

9. how much of the time during the PAST FOUR WEEKS have you felt calm and peaceful
__all of the time (1 points) __most of the time (2 points) __some of the time (3 points) __a little of the time (4 points) __none of the time (5 points)

10. how much of the time during the PAST FOUR WEEKS did you have a lot of energy
__all of the time (1 points) __most of the time (2 points) __some of the time (3 points) __a little of

the time (4 points) __none of the time (5 points)

11. how much of the time during the PAST FOUR WEEKS have you felt downhearted and depressed __all of the time (1 points) __most of the time (2 points) __some of the time (3 points) __a little of the time (4 points) __none of the time (5 points)

12. during the PAST FOUR WEEKS, how much of the time has your physical health or emotional problems interfered with your social activities (like visiting with friends, relatives, etc.) __all of the time (1 points) __most of the time (2 points) __some of the time (3 points) __a little of the time (4 points) __none of the time (5 points)
